# Characterization of thermally aged AlPO_4_-coated LiCoO_2 _thin films

**DOI:** 10.1186/1556-276X-7-12

**Published:** 2012-01-05

**Authors:** Eunhye Jung, Yong Joon Park

**Affiliations:** 1 Department of Advanced Materials Engineering, Kyonggi University, Suwon, Gyeonggi-do, 443-760, Republic of Korea

## Abstract

The electrochemical properties and stability during storage of pristine and AlPO_4_-coated LiCoO_2 _thin films were characterized. The wide and smooth surface of the thin film electrode might provide an opportunity for one to observe surface reactions with an electrolyte. The rate capability and cyclic performance of the LiCoO_2 _thin film were enhanced by AlPO_4 _surface coating. Based on secondary ion mass spectrometry analysis and scanning electron microscopy images of the surface, it was confirmed that the coating layer was successfully protected from the reactive electrolyte during storage at 90°C. In contrast, the surface of the pristine sample was severely damaged after storage.

## Introduction

Since Sony first developed lithium-ion batteries, extensive research has been carried out to obtain enhanced specific capacity, cyclic performance, and stability [1-4]. Surface coating of cathode powder is one of the most effective methods for improving the electrochemical property of lithium-ion batteries. As coating materials, oxides [5-8] and phosphates [8-11] have been employed to suppress unwanted interface reactions and enhance the electrochemical property of the cathode. However, the property of a coated cathode was highly dependent upon the coating material, coating thickness, and coating shape [12-15]. Hence, careful characterization of the coating layer of the cathode has been demanded. However, pristine cathode powders possess a circular shape with a diameter of several micrometers in general. After surface modification by coating, a number of nanosized particles attach to the surface of the pristine cathode powder. Thus, it has been difficult to characterize the coating layer directly because of the small particle size of the pristine powder, the very low thickness of the coating layer, and the rough surface of the positive electrode. Moreover, most techniques used to analyze the interface layer are seriously hampered by other components of the electrode, such as the binder and carbon.

Herein, the authors prepared an AlPO_4_-coated LiCoO_2 _thin film and investigated its electrochemical property. The LiCoO_2 _thin film electrode was introduced as a pristine cathode to investigate the intrinsic effect of AlPO_4 _coating on the electrochemical property. The smooth and wide surface of the thin film electrode proved to be good for characterizing surface reaction. In particular, this article focused on the effects of coating on rate capability, cyclic performance, and stability of the surface of the cathode film.

### Experimental details

The pristine LiCoO_2 _thin film was supplied by GS NanoTech Co., Ltd (Gangdong-gu, Seoul, South Korea). To prepare the coating solution, aluminum nitrate nonahydrate (Al(NO_3_)_3_9H_2_O; Sigma-Aldrich, St. Louis, MO, USA) and ammonium dihydrogen phosphate ((NH_4_)H_2_PO_4_; Sigma-Aldrich, St. Louis, MO, USA) were dissolved separately in 10 mL of a mixed solvent consisting of distilled water, 1-butanol, and acetic acid. Then, the solution was stirred continuously for 1 h at 25°C, after which the solution was coated onto a LiCoO_2 _thin film substrate using a spin coater (K-359 model S-1; supplied by Kyowa Riken Co., Ltd., Tokyo, Japan). The coated LiCoO_2 _thin films were heat-treated in a rapid thermal annealing system at 400°C for 30 min. The microstructures of the films were observed by field emission scanning electron microscopy (JEOL-JSM 6500F; JEOL Ltd., Tokyo, Japan). The electrochemical characterization of the coated LiCoO_2 _films was performed in nonaqueous half-cells. The cells were subjected to galvanostatic cycling using a WonAtech system (WonAtech Co., Ltd., Seoul, South Korea). Secondary ion mass spectrometry [SIMS] analysis was used to characterize both coated and pristine films to obtain the constituent element information. These characterization measurements were performed using a CAMECA IMS-6f (magnetic sector SIMS; CAMECA Company, Paris, France) at the Korea Basic Science Institute (Busan center). A Cs^+ ^primary ion beam with a beam energy of 5.0 kV was used. The beam current was 30 nA, and the raster size was 200 μm × 200 μm.

## Results and discussion

**Figure 1:**
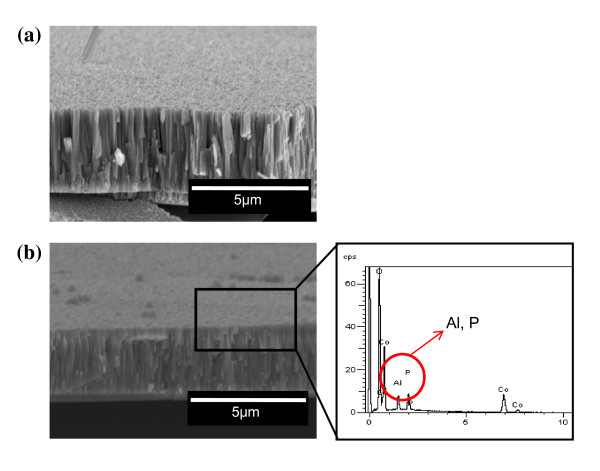
**SEM images of (a) Pristine and (b) AlPO_4_-coated LiCoO_2 _thin films**. The EDS peak of the coating layer is shown on the right.

**Figure 2:**
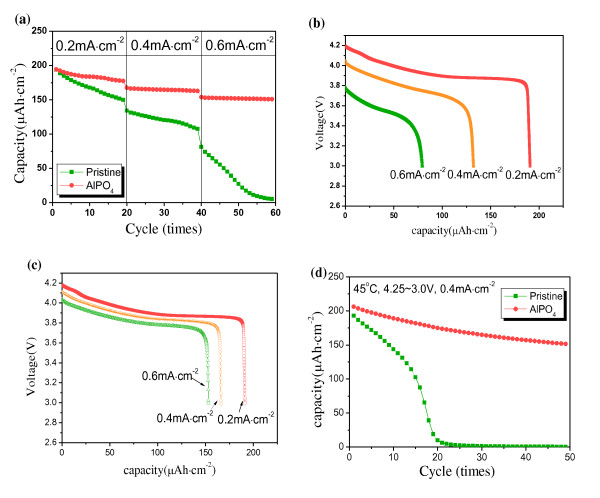
**The electrochemical properties of pristine and AlPO_4_-coated LiCoO_2 _thin films**. The films were measured in the voltage range of 4.25 to 3.0V. (**a**) Discharge capacities of the films at current densities of 0.2, 0.4, and 0.6 mA·cm^-2^. Initial discharge profiles of (**b**) pristine and (**c**) AlPO_4_-coated films (the first cycle of Figure 2a at each current density). (**d**) Cyclic performances of the films at 45°C (current density was 0.4 mA·cm^-2^).

The cross-sectional image of the pristine and AlPO_4_-coated LiCoO_2 _thin films is presented in Figure 1. The thickness of the LiCoO_2 _film was approximately 4 μm. The coating layer of the coated film was not clearly observed in the scanning electron microscopy [SEM] image, which may have been due to the thin coating thickness. However, the Al and P elements were confirmed using energy dispersive spectroscopy [EDS] analysis, which implied the existence of an AlPO_4 _coating layer. To confirm the coating effect on the electrochemical property, the discharge capacity and cyclic performance were measured at current densities of 0.2, 0.4, and 0.6 mA·cm^-2 ^in a voltage range of 4.25 to 3.0 V. The current densities could be converted to approximately 1-C, 2-C, and 3-C rates, considering that the average capacity of the LiCoO_2 _thin film is approximately 50 μAh·cm^-2^·μm^-1^. The film electrodes do not contain conducting agents, such as carbon, and they are vulnerable at the rate capability. As shown in Figure 2a, the initial discharge capacities of the pristine and AlPO_4_-coated samples were similar. However, with an increase in the current density, the coated film showed a superior discharge capacity and cyclic performance. Figures 2b and 2c present the voltage profiles of the pristine and coated samples at current densities of 0.2, 0.4, and 0.6 mA·cm^-2 ^as a function of the capacity (the first cycle of Figure 2a at each current density). The discharge capacity of the pristine film dropped rapidly at a high current density. However, the coated film showed much improved capacity retention under the identical condition.

The coating effect on the electrochemical property was very clearly observed during cycling at high temperature (45°C). The discharge capacity of the pristine film significantly decreased to nearly zero during 20 cycles (Figure 2d). Cyclic performance of the coated film also deteriorated compared with Figure 2a. However, the coated sample showed much enhanced cyclic performance over the pristine sample. The improved rate capability and cyclic performance of the coated sample have been explained by the protective effect of the coating layer. In general, the surface of the cathode forms an interface layer during cycling due to a reaction with the acidic electrolyte. The transition metals (Co, Ni, Mn) of the cathode could easily dissolve into the electrolyte so that the unwanted interface layer could be formed, which interrupts diffusion of the lithium ions and movement of electrons during cycling. A stable coating layer (AlPO_4_) could protect the surface of the cathode electrode from an attack by the acidic electrolyte and suppress the formation of an unwanted surface layer, leading to the enhanced rate capability and cyclic performance.

**Figure 3:**
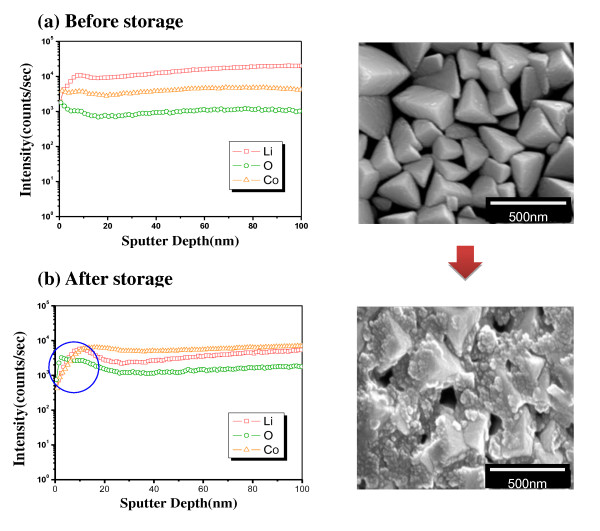
**Depth profile of the constituent elements and SEM surface image of the pristine LiCoO_2 _film**. (**a**) Before storage; (**b**) after storage.

**Figure 4:**
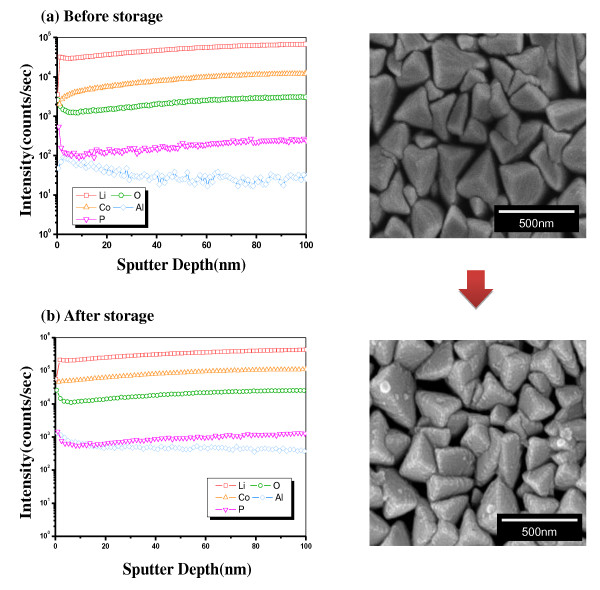
**Depth profile of the constituent elements and SEM surface image of the AlPO_4_-coated LiCoO_2 _film**. (**a**) Before storage; (**b**) after storage.

As a follow up test, the pristine and coated samples were charged to 4.25 V and stored at 90°C for 1 week to observe the protective effect of the AlPO_4 _coating layer under a harsh condition. The concentration profile (versus depth) of the pristine and coated films was measured by SIMS of the pristine sample. Co-containing ion (^133^Cs^59^Co^+^), O-containing ion (^133^Cs^16^O^+^), Li-containing ion (^133^Cs^7^Li^+^), Al-containing ion (^133^Cs^27^Al^+^), and P-containing ion (^133^Cs^31^P^+^) were recorded for the pristine and coated samples. As shown in Figure 3a, the pristine film consisted of a homogeneous concentration of Li, Co, and O, except for a small deviation in the surface prior to storage. However, it clearly shows that the Co was dissolved during storage, as shown in Figure 3b. The SEM image on the right also confirms the damage of the surface during storage. The surface of a LiCoO_2 _film showed clear polyhedral grains before storage. However, after storage, the surface of the grains was covered with small particles due potentially to a reaction with the electrolyte. In contrast, the coated film presented a very stable concentration profile and SEM images during storage (Figure 4). The AlPO_4 _coating layer was not clearly presented in the profile due to the high background intensity of the Al- and P-containing ions. However, it clearly shows that the Co concentration of the coated film was maintained stable after storage (Figure 4b), which confirms the protective effect of the coating layer from the reactive electrolyte. The SEM image also presents the stable surface of the AlPO_4_-coated film during storage. As shown in the right side of Figure 4a, the coated film was observed to possess a somewhat rough surface due to the coating layer. The noticeable point was that the surface grains of the coated sample were maintained without serious damage during storage. The stable concentration profile and SEM image of the AlPO_4_-coated film directly confirm the protective effect of a coating layer, which is associated with the enhanced electrochemical property of the coated electrode.

## Conclusions

An AlPO_4_-coated LiCoO_2 _thin film was characterized to investigate the surface coating effect. The AlPO_4_-coated film showed a superior rate capacity and cyclic performance over the pristine film. In the concentration profile (versus depth) of the pristine and coated films, it was clear that Co dissolution was successfully suppressed by applying an AlPO_4 _coating layer during storage at 90°C. The SEM image of the surface also shows the coated film to be stable from an attack of the reactive electrolyte during storage.

## Abbreviations

EDS: energy dispersive spectroscopy; SEM: scanning electron microscopy; SIMS: secondary ion mass spectrometry.

## Competing interests

The authors declare that they have no competing interests.

## Authors' contributions

EH did the synthetic and characteristic job in this journal. YJ gave the advice and guided the experiment.
